# Monitoring career impact and satisfaction in a graduate program in dentistry

**DOI:** 10.3389/fdmed.2025.1566272

**Published:** 2025-04-29

**Authors:** Isadora dos Santos Rotta, Fernando Valentim Bitencourt, Fabrício Mezzomo Collares, Roger Junges, Susana Maria Werner Samuel, Ramona Fernanda Ceriotti Toassi, Cassiano Kuchenbecker Rösing

**Affiliations:** ^1^ Department of Periodontology, School of Dentistry, Federal University of Rio Grande do Sul, Porto Alegre, Brazil; ^2^ Department of Dentistry and Oral Health, Section for Oral Ecology, Aarhus University, Aarhus, Denmark; ^3^ Department of Dental Materials, School of Dentistry, Federal University of Rio Grande do Sul, Porto Alegre, Brazil; ^4^ Institute of Oral Biology, Faculty of Dentistry, University of Oslo, Oslo, Norway; ^5^ Department of Preventive and Social Dentistry, School of Dentistry, Federal University of Rio Grande do Sul, Porto Alegre, Brazil

**Keywords:** graduate education, specialization, continuing dental education, dental education, cross-sectional studies, clinical competence

## Abstract

**Introduction:**

The assessment of student outcomes is essential for monitoring the quality of graduate programs in healthcare sciences. As such, this study focused on developing a self-employed questionnaire that allowed for the evaluation of elements focused on career impact and levels of satisfaction regarding graduate program education. Following, this instrument was utilized in a cross-sectional study design with alumni that had obtained their degree (MSc or PhD) over a 25-year span (1995–2020) from a graduate program in dentistry located in Brazil.

**Methods:**

The employed instrument comprised a total of 43 questions presenting a mix of both close and open-ended questions coupled with 5-point Likert scales. The questionnaire was hosted online and a total of 528 alumni were invited to participate through e-mail and social media outreach.

**Results:**

376 alumni answered the questionnaire (71.2% response rate). The majority were female (69.9%), and with a MSc (58.5%). Levels of satisfaction towards the program as well the impact in career and life were higher in alumni that had obtained a PhD degree compared to MSc. After obtaining the degree, an increase in involvement in teaching/research positions (3.4% vs 21.5%, *p* < 001) and a decrease in unemployment (21.9% vs 2.1%, *p* < 001) were observed. The highest levels of impact were observed regarding the achievement of the professional goals as nearly 90% of the population agreed with this statement.

**Conclusions:**

This study highlighted the creation and employment of an assessment tool that can be utilized to monitor the perceptions of student outcomes. Among the findings, a decrease in unemployment and a high degree of career impact and satisfaction were observed in the population of this study. Moving forward, it is essential that monitoring educational outcomes remains a priority worldwide.

## 1 Introduction

Higher educational levels in the population are directly correlated with better quality of life and a higher human development index (1). Graduate programs worldwide comprise different systems with varied guidelines and modes of functioning. For example, doctoral programs (often used as a synonym of PhD) in dentistry are often inserted as a “third cycle” after bachelor's and master's (MSc) degrees (2). However, there are also programs that allow students to partake their MSc or PhD training while they are also obtaining their dental degree (3). In all these contexts, PhD training is seen as an important element in educating professionals equipped to work with research/development and that are interested in an academic career (4). In the context of the present article, a doctoral program is referred to as a teaching/research-based PhD program. Further, evaluating the impact of pursuing a graduate degree on student outcomes is crucial for the continuing development of such programs and for the development of policies governing agenda of graduate programs both internationally and locally.

Brazil stretches over a large territory with over 600 dental schools and over 100 graduate programs focused on providing students with the opportunity to obtain a MSc and/or a PhD degree. These programs are often research-focused and aim at generating new knowledge while also providing human resources for teaching/research positions. The institutionalization of graduate programs in Brazil was based in Parecer Sucupira (5). This document led to the constitution of a robust scientific community and received the first incentive in the second half of the 1960s (6). Between 1998 and 2020, an increasing number of academic programs was observed in the country (7). Similarly, academic graduate programs in dentistry increased, and over 100 teaching/research-focused graduate programs are currently offered nationwide (8). The evaluation of academic graduate programs in Brazil is carried out by Coordination for the Improvement of Higher Education Personnel (CAPES). This process was boosted in the 1990s and has continuously improved over the years. Currently, the evaluation process is based on a multidimensional approach. Among the guidelines, analysis of the quality of education of MSc and PhD degrees and emphasis on items that intend to differentiate the quality of the different programs are currently incorporated (8).

The monitoring of alumni is a strategy that allows for the understanding of aspects related to the educational process. As such, data regarding employment and the identification of skills, strengths and weaknesses in the program are relevant for their assessment (9, 10). Assessing alumni satisfaction is a key factor influencing the success and quality of a higher education programs, as it serves as an indicator of teaching and learning and assists universities to improve their processes (11, 12). In addition, analyzing the perceptions of alumni supports internal reevaluation/restructuring and provides data on students' expectations and needs (13) so that they can be better prepared to face the job market (14). Several studies have evaluated student's profile, career decisions, expectations and job market assignments after obtaining a dental degree (10, 15–17). For example, in a study conducted with 945 recently graduated dentists in the Netherlands, over 50% expected owning their own practices within 5 years of graduation (17). In a 10-year prospective study including final-year dental students, 20% expressed intention to pursue a MSc and PhD degree after graduation. Also, 53% intended to pursue a specialization/residency course, that focused on specialized training (15). In a national survey of final-year undergraduate students in the United Kingdom, students revealed key points of overall satisfaction such as quality of teaching, level of support, institutional organization, staff availability (18).

Assessment tools that allow for the standardized evaluation of such aspects in graduate programs in dentistry are lacking. Consequently, studies assessing the career paths of alumni from academic graduate programs (especially focused on teaching/research), including their personal and professional satisfaction, are still scarce. Given the relevance of analyzing the achieved results of the educational process in graduate teaching/research programs, this study developed a self-employed questionnaire that allows for the evaluation of the perceptions of alumni from graduate programs in dentistry, with an aim to assess career impact and levels of satisfaction regarding the program. The tool was employed to collect responses from alumni that had obtained their degree (either or both MSc or PhD) over a 25-year span (1995–2020) from a graduate program in dentistry in Brazil, focused on five main domains: socio-demographic profile, work experience and perceptions regarding education, career impact, scientific productivity, and future perspectives.

## 2 Materials and methods

### 2.1 Ethical considerations

Ethical approval was obtained by the Institutional Review Board of the Federal University of Rio Grande do Sul (approval number 4.255.668**/**CAAE: 03448212.6.0000.5347). Participants agreed to participate in this study through the informed consent form, which was embedded into the online questionnaire. All data were treated confidentially.

### 2.2 Questionnaire design and validation

The questionnaire was developed focused on a multidimensional framework, based on the technical report by CAPES (19), which outlines relevant key areas regarding the assessment of graduate programs. Content and face validation were employed as the instrument was created by a panel of experts from multiple disciplines based on extensive literature searches and reviewed by five professors from the institution under study, with expertise in questionnaire development, higher education, and research methodology. Following, a pilot study was conducted with ten volunteer students from graduate educational programs elsewhere to assess clarity, relevance, internal consistency of the proposed instrument and identify challenges that could be faced in the administration of this assessment tool (Cronbach's Alpha *α* = 0.908).

The final instrument consisted of 40 closed and 3 open-ended questions (Supplementary Material) organized into five dimensions: (1) socio-demographic profile, (2) work experience and perceptions regarding education, (3) career impact, (4) scientific productivity, and (5) future perspectives (Table 1). Each section was designed to comprehensively address one or more specific domains of the CAPES assessment framework. Data was collected via *Google Forms* (Google; Mountain View, CA, USA).

**Table 1:** Structure of the questionnaire dimensions.

Dimensions	Description of data collected	Number of items
Participant context (socio-demographic profile)	Sex; age; marital status; city/state/country of origin; current city/state/country; academic educational profile (location and year of DDS degree, participation in research during DDS degree, involvement in graduate studies: specialization/residency course, master degree and/or doctoral degree).	13
Work experience and perceptions regarding education	Employment status prior to obtaining the graduate degree; employment status during the course; weekly time allocation to the program, multidisciplinarity and internationalization of the research project; entrepreneurship initiative; further education in other institutions; satisfaction levels towards the degree development.	12
Career impact	Employment status after obtaining the graduate degree(s), teaching involvement and at which level, involvement in management positions; and perceived career impact of the academic graduate education.	10
Scientific productivity	Status of the dissertation/thesis in terms of publication; and academic productivity during/after obtaining the graduate degree.	3
Future perspectives	Future perspectives and recommendation of the graduate program to colleagues and/or extended network.	5

A 5-point Likert scale was used to access parameters of satisfaction with a range of components in the graduate program and the impact on the career of alumni. Perceived career impact was explored by the following criteria: employability and wages, professional growth and social benefits. Satisfaction was assessed by program management, curriculum structure, external activities, language exposure opportunities, infrastructure for teaching and research, academic support, faculty members profile, and mobility opportunities. The respondents were asked to indicate their level of agreement with each topic of satisfaction and perceived impact, including a scale from 1 (strongly disagree or very poor) to 5 (strongly agree or very good). Higher scores reflect greater levels of satisfaction with the program and perceived impact. They were also provided with an open-ended question given the opportunity to provide free text comments to the question, “Describe how you perceive the impact of the degree obtained on your life, in personal, professional, and academic dimensions.”.

### 2.3 Study design and context

A cross-sectional questionnaire-based study was conducted among alumni of the Graduate Program in Dentistry from the Federal University of Rio Grande do Sul (UFRGS), a public university in the south of Brazil. The program was established in 1991 and recognized by CAPES. Currently, it offers both MSc and PhD programs in dentistry, with three research-focus areas: Dental Clinics, Oral Pathology, and Community Dentistry. It is composed by over 40 affiliated faculty members and aims to develop qualified professionals with a focus on academic and research skills. Following the CAPES evaluation scale, ranging from 1 to 7, it was evaluated with a score 6. This score defined the graduate program as high-quality with international status (criteria: number of articles published, number and sum of obtained grants, highest level of education of affiliated faculty, among others).

### 2.4 Sampling and inclusion/exclusion criteria

As inclusion criteria, all the 528 alumni who obtained a master's and/or doctoral degrees between 1995 and 2020 were invited to participate. Access to records of contact information of all participants was performed in collaboration with the administrative staff of the graduate program. E-mail invitation to participate with a link to the survey was sent twice to participants, with an interval of fifteen days between each. Concomitantly, two researchers (ISR and FVB) conducted searches through social media platforms to interact and establish contact with those alumni whose e-mail addresses were unresponsive. The data collection phase took place between September and November 2020.

### 2.5 Statistical analysis

Data analysis was performed using a statistical package (SPSS version 24.0, SPSS Inc., Chicago, IL, USA). Absolute values were used for statistical testing. Chi-square test was used for comparing employment and data from Likert scales. Further, considering that it is challenging to assume that the distance between the sequential options on a Likert scale are equal (e.g., very poor, poor, acceptable, good, very good) (20), data were described as collected to avoid potential bias in interpretation. Alumni who completed an MSc and a PhD, were categorized into the PhD group.

## 3 Results

### 3.1 Socio-demographic profile

In total, 376 participants answered the questionnaire, yielding a response rate of 71.2%. Socio-demographic characteristics of the study population are available in Table 2. Prior to enrolment in the graduate program, 48.7% of participants (*n* = 183) had also obtained their Doctor of Dental Surgery (DDS) degree in the same university. Concerning the location of the university in which the participants underwent their dental degree studies, the majority (94.1%, *n* = 354) was in Brazil and the following regions: South (86.7%, *n* = 326), Southeast (2.9%, *n* = 11), Midwest (1.3%, *n* = 5), North (1.3%, *n* = 5), Northeast (1.9%, *n* = 7). A total of 16 students (4.3%) obtained their dental degrees abroad. Close to one-third (30.1%) of the participants in the survey that undertook their MSc studies continued to a PhD degree in the same institution. Conversely, 12.7% pursued a PhD degree in a different institution. An increasing proportion of alumni that were part of a research program during their undergraduate education was observed, moving from 33.8% of participants who obtained their DDS during 1991–2000, to 42.1% in 2001–2010, and 59.5% for those who graduated in 2011–2020 (data not shown).

**Table 2:** Socio-demographics and education characteristics for MSc and PhD alumni from 1995 to 2020.

Variable	Frequency
	n (%)
Sex	
Female	263 (69.9)
Male	113 (30.1)
Age (years)	
24–33	149 (39.6)
34–43	147 (39.1)
44–53	61 (16.2)
54–63	11 (2.9)
64–74	7 (1.9)
Not available	1 (0.3)
Academic graduate degree obtained	
Master degree (MSc)	220 (58.5)
Doctoral degree (PhD)	156 (41.5)
Year of undergraduate – DDS degree	
1971–1980	4 (1.0)
1981–1990	12 (3.2)
1991–2000	71 (18.9)
2001–2010	121 (32.2)
2011–2020	168 (44.7)
Participation in a research program during DDS degree	
Yes	178 (47.3)
No	198 (52.7)
Specialization studies or residency (completed or ongoing)	
Yes	240 (63.8)
Specialization	198 (52.6)
Residency	23 (6.1)
Specialization and residency	19 (5.1)
No	136 (36.2)

### 3.2 Work experience and perceptions regarding education

**Figure 1:**
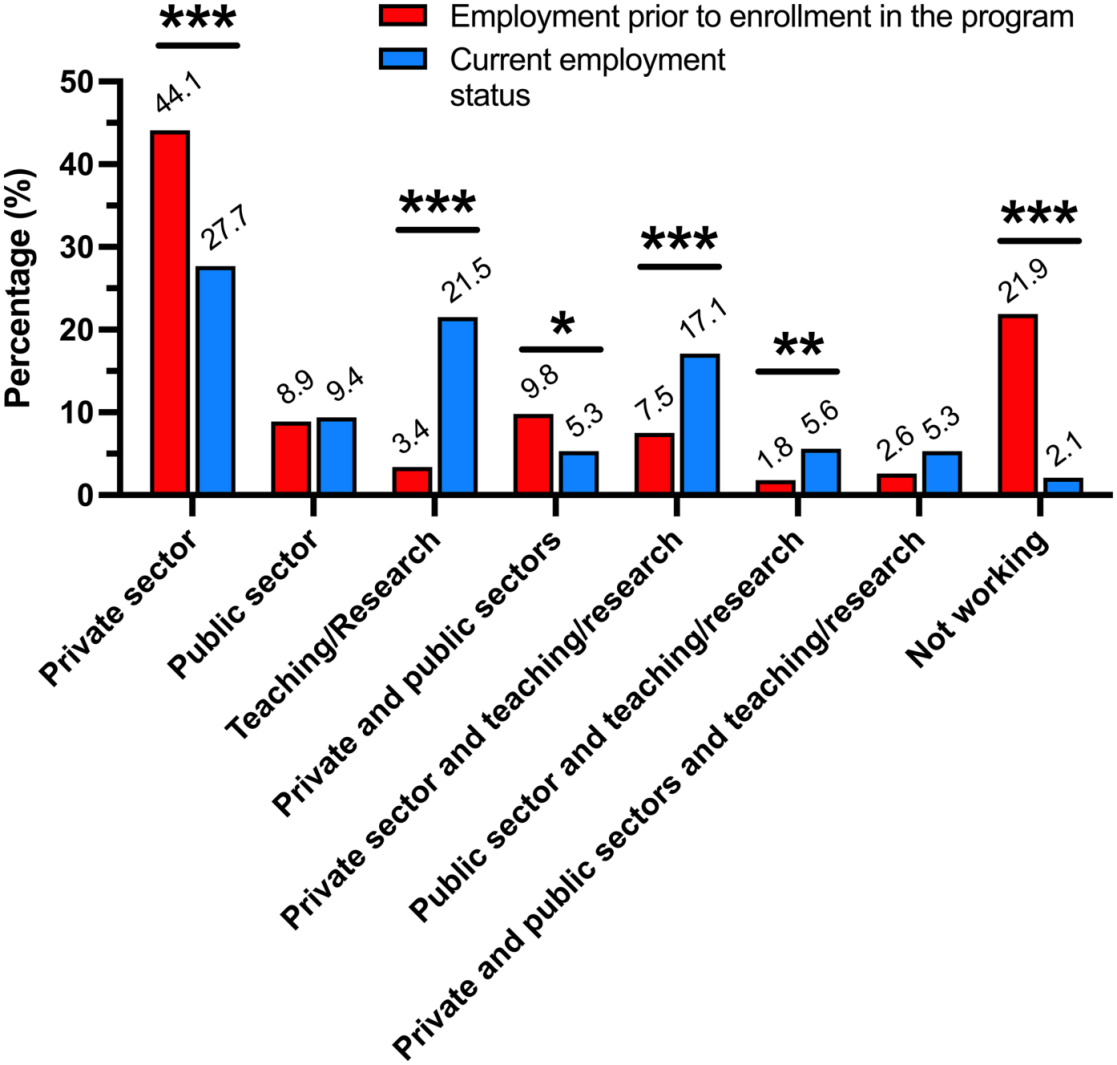
Employment status prior to enrollment in the graduate program in parallel with current employment. Red bars represent employment prior to program enrollment, while blue bars indicate current employment as of 2020. Bars represent the total count of responses for each category. In addition to the “current employment” responses shown in the figure, 21 participants indicated to be currently enrolled in a graduate program, 1 participant has changed profession, and 1 has retired. *p < .05, **p < .01, ***p < .001.

**Figure 2:**
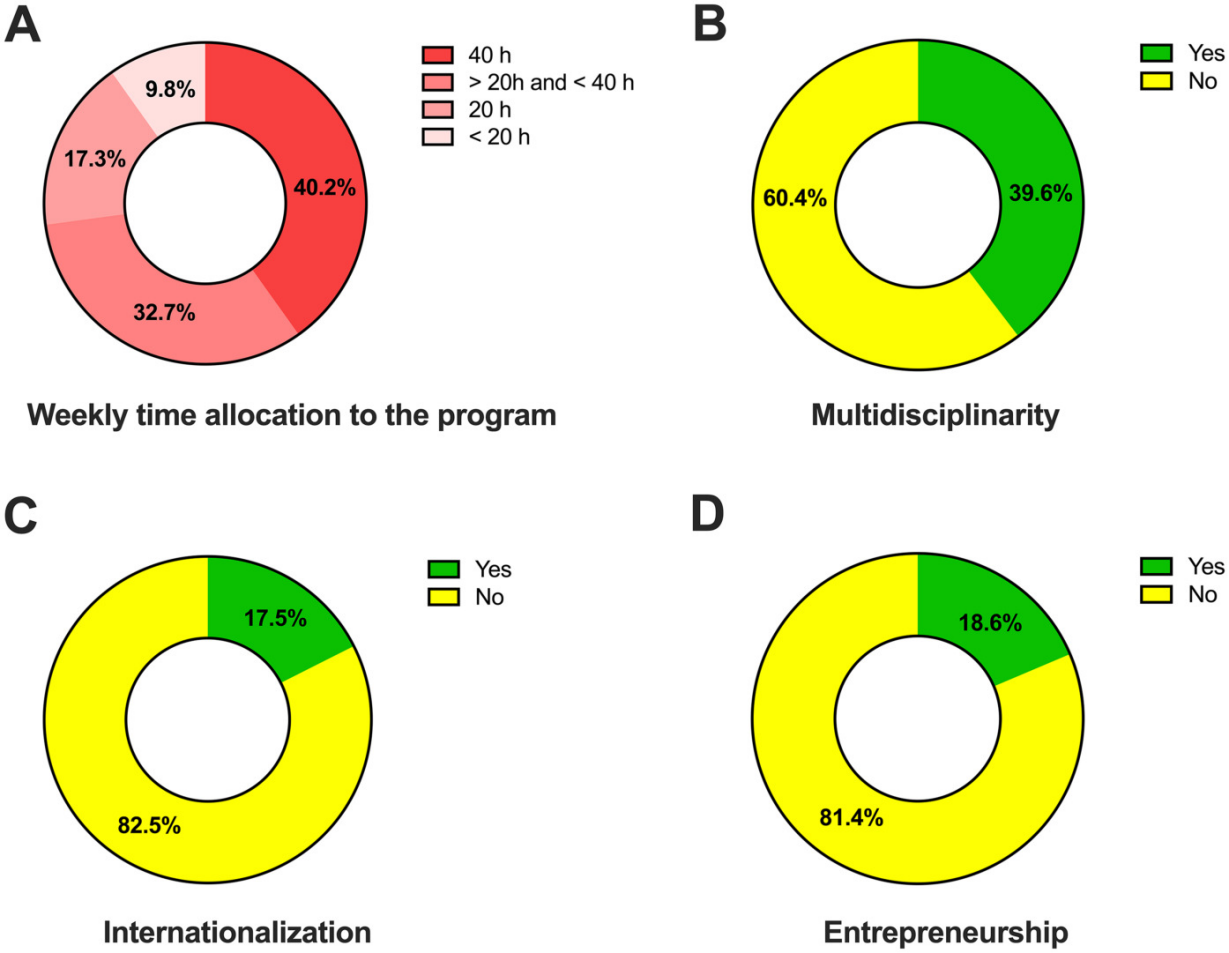
Research project characteristics and time allocation. **(A)** Weekly time allocation by the alumni to the program. **(B)** Multidisciplinary approach in the research project. **(C)** Interactions with research groups abroad. **(D)** Engagement in entrepreneurship after graduate degree.

Work experience prior to enrollment in the graduate program in comparison with current employment status is shown in Figure 1. An increased number of participants involved with teaching/research positions after completing the graduate degree was observed (3.4% vs. 21.5%, *p* < 001). Also, a decrease in the percentage rate of individuals unemployed (21.9% vs. 2.1%, *p* < 001) were observed. Figure 2A shows weekly time dedication to the program. Further, participants reported multidisciplinary in the project (Figure 2B) and interaction with research groups abroad (Figure 2C). When interacting with other scientific fields, the areas reported were within healthcare sciences (43.1%), biological sciences (22.8%), engineering (12.6%), exact sciences (10.6%), human sciences (7.2%), agricultural sciences (2.4%), and social sciences (1.2%) (data not shown).

**Figure 3:**
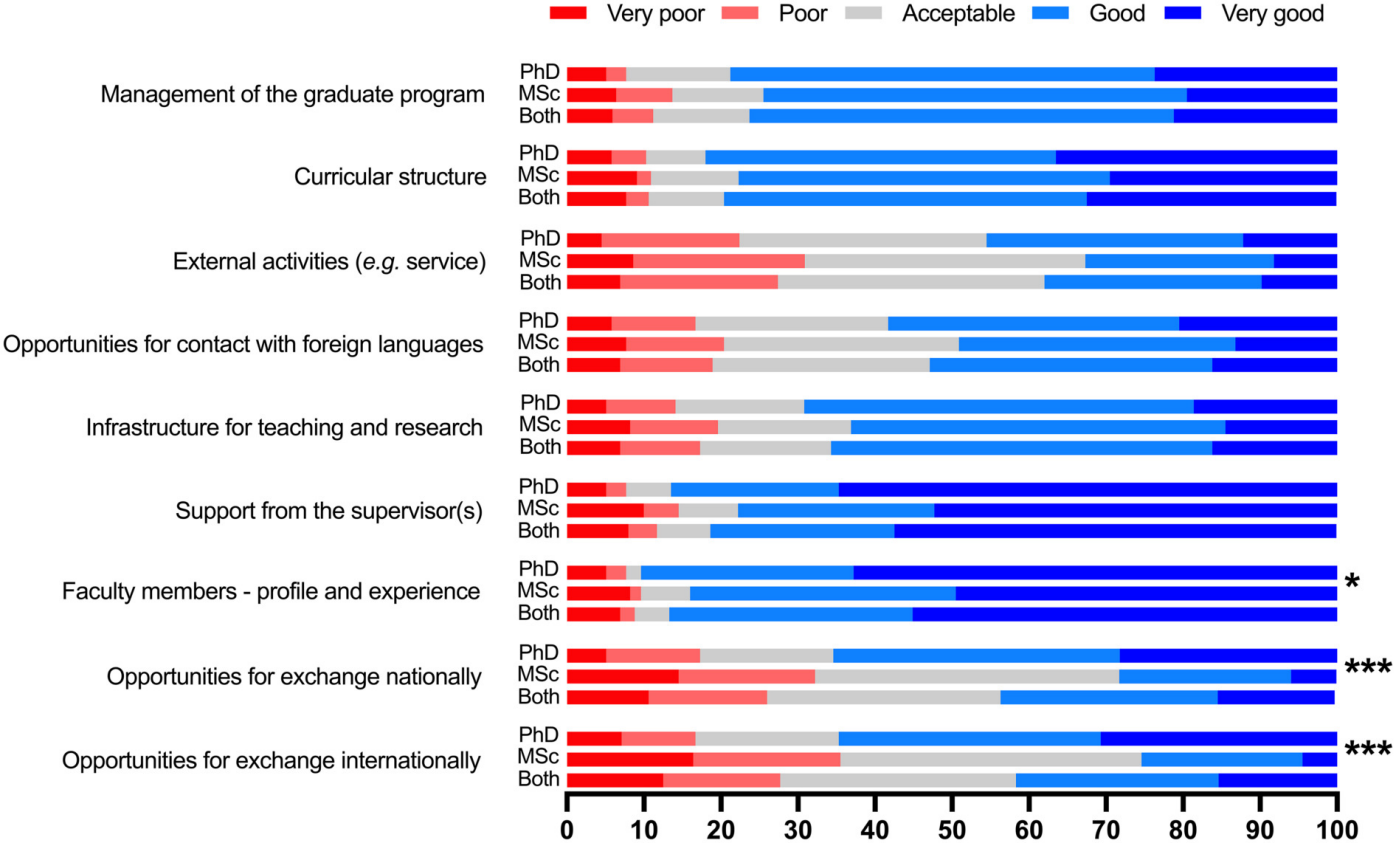
Satisfaction levels of MSc and PhD alumni from 1995 to 2020. Nine different items were proposed, and participants were asked to select their level of agreement in a 5-point Likert scale regarding the level of quality of each proposed topic regarding the program. Comparison between alumni’ from the MSc and PhD programs; Chi-square test. *p < .05, ***p < .001.

For 70 (18.6%, Figure 2D) participants that reported being engaged in entrepreneurship after conclusion of their graduate courses, 38.6% (*n* = 27) expressed that the program was “Extremely important” for such, 21.4% (*n* = 15) “Very important”, 11.4% (*n* = 8) “Of average importance”, 15.7% (*n* = 11) “Of little importance”, 12.8% (*n* = 9) “Not important at all”. With regards to further education, 67 participants (17.8%) reported to having pursued a PhD (*n* = 32, 8.5%), a postdoc (*n* = 25, 6.6%), or both (*n* = 10, 2.7%) in other educational and/or research institutions. Countries such as Australia, Austria, Brazil, China, England, France, Germany, Netherlands, Japan, Sweden, the United States of America, and Uruguay are among the places considered for continuing education (data not shown). Further, levels of satisfaction with a variety of the components in the programs are presented in Figure 3. “Faculty members—profile and experience” was the item rated most positively by both MSc and PhD alumni. No significant differences were found between MSc and PhD alumni regarding program management, curriculum structure, external activities, language exposure, infrastructure, and academic support.

### 3.3 Career impact

**Figure 4:**
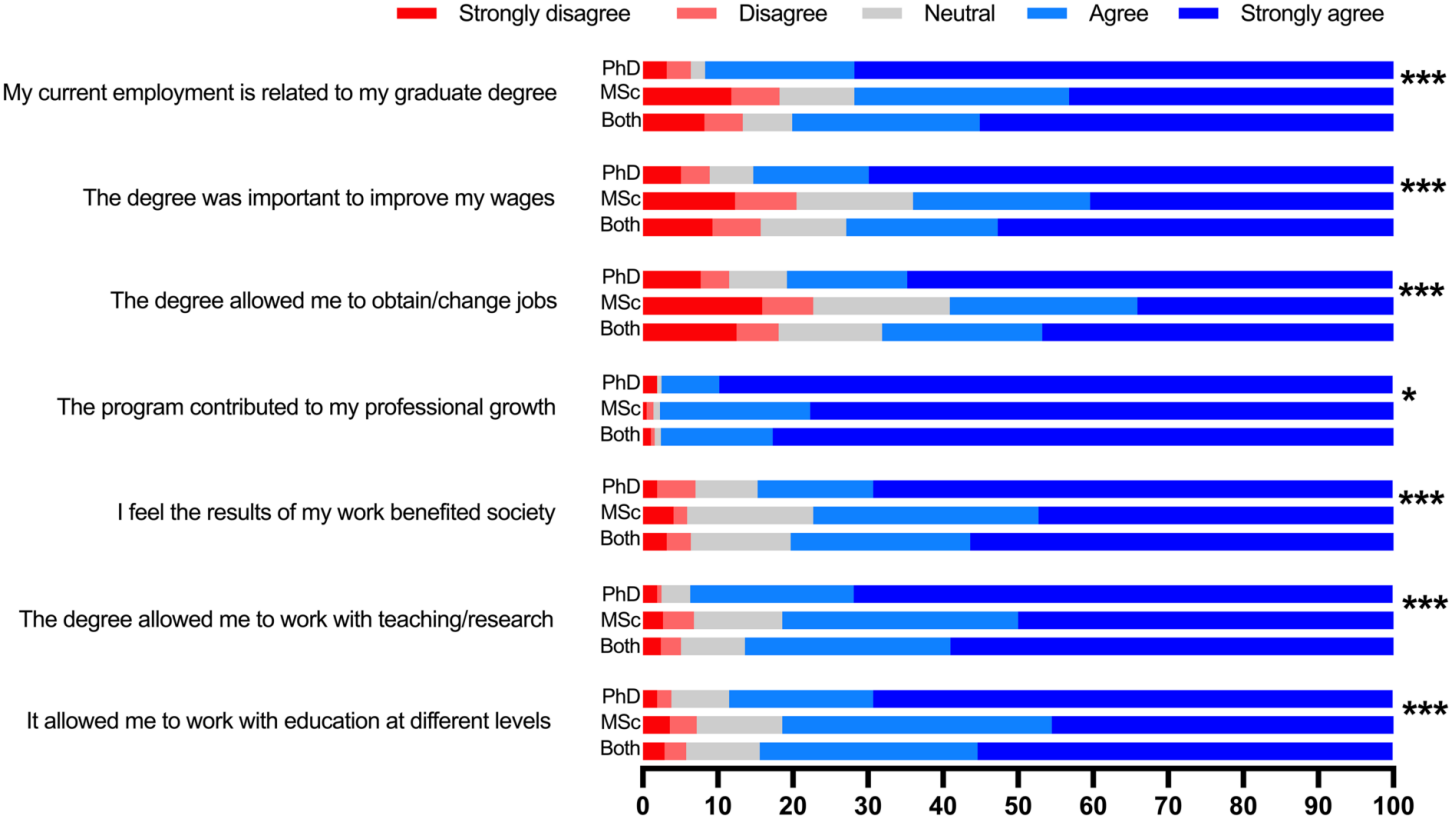
Perceived impact of the program in the careers of MSc and PhD alumni from 1995 to 2020. Seven different statements were provided to participants, who were asked to select their level of agreement in a 5-point Likert scale. Comparison between alumni’ from the MSc and PhD programs; Chi-square test. *p < .05, ***p < .001.

As described in Figure 1, a significant increase in the involvement of alumni in teaching/research positions was observed. With regards to the level of teaching, 195 participants (51.9%) declared to have been involved with undergraduate dental students, 171 (45.5%) in specialist training/residency programs, and 61 (16.2%) in MSc and/or PhD programs. A total of 151 (40.1%) reported to be involved in management positions ranging from local to national institutions, and also abroad. Figure 4 shows responses regarding the perceived impact of the program in their lives and careers. Overall, the perceived impact was consistently higher among PhD alumni across all factors when compared to MSc alumni. Among all evaluated items, the slightest difference between PhD and MSc alumni was identified in their agreement to: “The program contributed to my professional growth”.

### 3.4 Scientific productivity

Participants were asked whether the results of their dissertation/thesis had been published, and 224 (59.6%) reported having published it, while 100 (26.6%) indicated to be in the process of publication. The number and rate of participants that reported to not having published their results was higher for individuals that had only taken part in the MSc program (*n* = 39, 17.7%) compared to individuals involved in the PhD program (*n* = 13, 8.3%) (data not shown). Table 3 shows the frequency of participants that reported publication for different items. For articles published in international journals, a higher frequency was observed for students that interacted or collaborated with research groups abroad (83.3 vs. 66.1%, *p* < .01) or that worked on multidisciplinary projects (75.8 vs. 64.8%, *p* < .05) (data not shown). Concerning collaborations, 16% reported to have a publication with international collaborators, 18.4% with national collaborators, and 2.4% with industry partners.

**Table 3:** Frequency of different types of academic productivity during and after the conclusion of the graduate research program.

Academic producticity	Frequency n (%)		
	MSc (*n* = 220)	PhD (*n* = 156)	Total (*n* = 376)
Scientific articles			
Article published in international journals	130 (59.1)	130 (83.3)	260 (69.1)
Article published in national journals	108 (49.1)	121 (77.6)	229 (60.9)
Book or book chapter published internationally	4 (1.8)	20 (12.8)	24 (6.4)
Book or book chapter published nationally	31 (14.1)	67 (42.9)	98 (26.1)
Scientifics meetings			
Abstract published in international meetings	75 (34.1)	110 (70.5)	185 (49.2)
Abstract published in national meetings	134 (60.9)	130 (83.3)	264 (70.2)
Study published in the proceedings of international meetings	30 (13.6)	42 (26.9)	72 (19.1)
Study published in the proceedings of national meetings	52 (23.6)	53 (34.0)	105 (27.9)
Presentations			
Symposium or keynote speaker	32 (14.5)	55 (35.2)	87 (23.1)
Invited talks	69 (31.4)	89 (57.0)	158 (42.0)
Presentation in scientific meetings	156 (70.9)	130 (83.3)	286 (76.1)
Lecturer in further education courses	33 (15.0)	56 (35.9)	89 (23.7)
Other types of productivity			
Patents	6 (2.7)	11 (7.0)	17 (4.5)
Newsletters	3 (1.4)	9 (5.8)	12 (3.2)
Technical standards	5 (2.3)	3 (1.9)	8 (2.1)
Technical work	1 (0.4)	2 (1.3)	3 (0.8)
Translation	1 (0.4)	0 (0)	1 (0.3)

### 3.5 Future perspectives

Table 4 shows results indicating the future perspectives of alumni from the program. When questioned if they would recommend the MSc and/or PhD programs in their professional network, 251 (66.8%) answered “Absolutely”, 94 (25%) stated “Yes”, 25 (6.6%) responded “Maybe”, 5 (1.3%) “No”, and 1 (0.3%) “Absolutely not” (data not shown).

**Table 4:** Future perspectives of the participants in the survey. Participants could select more than one option. Percentages are calculated based on the number of respondents in each educational group (MSc or PhD).

Future perspectives	Frequency n (%)		
	MSc (*n* = 220)	PhD (*n* = 156)	Total (*n* = 376)
Employment			
Private sector	131 (59.5)	56 (35.9)	187 (49.7)
Public sector	64 (29.1)	41 (26.3)	105 (27.9)
Teaching	68 (30.9)	105 (67.3)	173 (46)
Research	53 (24.1)	90 (57.7)	143 (38.0)
Career in teaching/research			
Apply for teaching/research positions	69 (31.4)	43 (27.6)	112 (29.8)
No plans of pursuing a teaching/research career	22 (10.0)	4 (2.6)	26 (6.9)
Further education			
Pursue PhD studies in Brazil	96 (43.6)	3 (1.9)	99 (26.3)
Pursue PhD studies abroad	28 (12.7)	4 (2.6)	32 (8.5)
Pursue a postdoc in Brazil	17 (7.7)	44 (28.2)	61 (16.2)
Pursue a postdoc abroad	19 (8.6)	56 (35.9)	75 (19.9)
Other	7 (3.2)	4 (2.6)	11 (2.9)

## 4 Discussion

This study developed an assessment tool that could monitor the perceptions of alumni from graduate programs in dentistry with a focus on career impact and satisfaction. Further, this instrument was utilized to investigate the perceptions of 376 alumni from a graduate program in dentistry located in Brazil. As such, a variety of factors such as curricular structure, facilities, and the experience of faculty members were evaluated. Due to the relatively high response rate, the findings presented here are thought to be representative perceptions of the referred study population. As previously stated, the graduate program under study is academic, focused in research and teaching. Employment in teaching/research positions was significantly higher after receiving either MSc or PhD degree, given that the graduate program lays a solid training on research and teaching. In this sense, job opportunities increased for the graduates under study. Overall, we identified that participants showed a high level of satisfaction towards the program and a perceived high impact in their careers as a result of acquiring the graduate academic degree. In general, levels of satisfaction and perceived impact were more pronounced in participants who obtained a PhD degree as compared to those receiving the MSc degree.

A strong perceived career impact of the graduate research program was observed in several aspects that were evaluated in this survey. Areas that were measured ranged from employability to professional growth, and benefits to society. In all factors considered, PhD alumni reported a higher perceived impact compared to MSc alumni. Such differences have been observed previously (14) and can be interpreted as a possible result of various factors such as time spent in the program, complexity-reward of the research project, and career seniority (21, 22). In addition to the fact that a PhD degree is the highest academic degree that can be obtained, it is generally seen as more impactful than a research MSc degree. Despite being slightly lower than the PhD degree, the positive impact observed by MSc alumni was higher than 50% for all points listed. Considering all participants, the two highest impacts perceived were related to professional growth and capacity building to work in teaching/research, which aligns with the core goals of graduate education (5, 19). Our findings go in line with previous reports from a research-intensive program in Canada, in which majority of graduates indicated that their current work relied on their academic skills developed during the program (14). In this study, satisfaction assessment was a key component of the evaluation. Our instrument included nine program-related components. Although our instrument was based on the local context, and the different approaches to assessing satisfaction, it aligns with the literature regarding crucial evaluation aspects, such as program management, curriculum structure, mobility, academic support, among others (12, 23). The UK National Student Survey employs a tool that encompasses six main areas, with one being “assessment and feedback”, which was not included in our instrument. Despite this limitation, this evaluation may not yield significant additional insights, as it has been considered a poor predictor of overall satisfaction measurement (12).

The highest satisfaction point was related to the profile and experience of the faculty members. Most faculty members present a track record of successful funding and publication history and are part of national/international research networks. Previous studies have reported high satisfaction rates with the education for both undergraduate and graduate students, which go in accordance with our findings particularly regarding the profile of faculty members and the availability of resources (13, 24–26). Significant differences between PhD and MSc alumni were observed for exchange opportunities (both nationally and internationally). As MSc degrees are often obtained in approximately two years, the complexity of the research project and the funding are not always permissive to research stays in other institutions, which, on the other hand, is one of the priorities for PhD students. Even though this finding is expected, the possibility of creating more opportunities tailored for MSc students in dentistry should be further investigated. Results from a successful program in Europe showed the unique added value of such opportunities and can be helpful when designing new strategies (27).

The involvement of undergraduate students in research is a priority in dental education in Brazil. Skills such as critical thinking and the comprehension of the scientific process can be enhanced by engaging students with research early in the studies (28–31). A recent study observed many positive effects of involving dental students in research, which were observed not only in students' metrics but also in the whole academic environment (28). We have found that a higher rate of students engaged in a research program was observed over time. The involvement of students with research should be seen as a priority worldwide as it can enhance their skills for future employment, increased scientific publications and quality, and improvement in the dissemination potential of science in society (29). Coupled with specific time allocation in dental curricula, public and private incentives such as scholarships and research grants can have a powerful impact in directing these trends and should be seen as a priority for funding agencies and public policies (28, 32).

The employment of alumni is one of the central aspects of obtaining a graduate degree (14, 22, 33). In the results of this study, a high percentage rate of unemployed participants (21.9%) prior to starting the course was observed and a significant reduction followed the completion. This can be partially explained by the high number of recent dental graduates who wished to enroll in a graduate course. Competition in Brazil is high, and dentists perceive a necessity to pursue continuing education to establish themselves in the market, particularly early in their careers. Thus, even though a positive decrease was observed, the initial unemployment rate might have been higher than expected. In addition, another potential explanation is that some participants were graduate students during the survey and did not regard it as formal employment. Similar findings have been observed in a recent survey assessing dental undergraduates' employment patterns and graduate education (10). Applicants with a PhD degree have a competitive advantage, which can be reflected in employment patterns for teaching/research positions and perceptions (14).

One of the strong points in this study is the high response rate for an online survey, which is indicative that the sample represents the study population. In addition, the inclusion of alumni from all cohorts since the program's beginning enhances the data by incorporating a wide range of perceptions regarding the learned experiences. However, this picture presented here is a cross-sectional survey and covers a 25-year time span (1995–2020), which may have introduced variation in alumni responses due to differences in program structure, context, professional individual experiences over time. Caution should be exercised when interpretating the data. Despite the relatively high response rate and the cross-sectional study design, the non-response rate bias should be considered (34). A longitudinal design that closely monitors MSc and PhD alumni from the beginning of their studies through their professional careers is currently lacking, yet it would significantly enhance the understanding of graduate programs and support institutional planning. Close assessment and follow-up are one of the priorities of educational programs in order to help shape the institution's future (21, 22). We have created a tool applicable to the context in this study following elements that are important for instrument construction such as content and face validity; however, caution is necessary given that for a more comprehensive approach further validation is necessary. Such steps would also assist in the potential utilization of the tool in different diverse contexts. Further, an assessment score successfully encompassing all the domains proposed would facilitate the evaluation of changes over time and before/after introduction of changes in the programs.

While this study evaluated the program as a whole, it was outside of the scope to assess how demographic data of the participants correlates with perception over the program or perform a temporal analysis. The evaluation tool was constructed based on key dimensions to assessing graduate programs and also included qualitative indicators for the evaluation process. The tool focused on capturing alumni perceptions and satisfaction regarding their experiences in graduate education. In the future, assessments should also include more targeted indicators of research competency including production but also beyond publication output. These could involve evaluating skills in research innovation, entrepreneurship, the application of community-based research to improve health in diverse populations, and engagement in national and international collaborations. Integrating research more fully into graduate dental education is essential not only to strengthen academic and scientific training, but also to ensure that evidence-based knowledge is effectively translated into clinical practice (35, 36). Further, there are a variety of individual characteristics that may vary significantly between participants and are elusive to assess, such as the interaction with the research group and supervisor, which were not in the scope of this study but are important to assess in future (37–39). Of note, albeit positive, the results should be interpreted with caution as they are self-reported data and can present bias (40). Furthermore, analyses of the qualitative components of the questionnaire are currently underway and will add to the quantitative results presented here.

The overall findings in this study are promising as the alumni perceived a strong contribution of the graduate program in their careers and lives. Also, the respondents of this survey demonstrated good levels of satisfaction with their degree. Moreover, the results identify an increase in employment after the degree is obtained and a higher engagement in teaching/research positions. Even though program, country- and region-specific characteristics shape the job market, disseminating findings on graduate program assessment strengthens our understanding of how best to provide continuing education to dental professionals. A standardized assessment tool, like the one developed in this study, enables cross-sectional and longitudinal alumni evaluations. While designed for a graduate program in dentistry, the instrument can be adapted and employed in other settings, considering differences in curriculum, institutional reputation and personal experiences. Ongoing data collection will further refine future iterations for alumni assessment.

## Data Availability

The raw data supporting the conclusions of this article will be made available by the authors, without undue reservation.
