# Supplementary material for: Monitoring career impact and satisfaction in a graduate program in dentistry
Source: Front Dent Med. 2025 Apr 29;6:1566272. doi: 10.3389/fdmed.2025.1566272 (PMC12069376; doi:10.3389/fdmed.2025.1566272)
Supplement: Supplementary file 1 [file Datasheet1.docx]

Supplementary Material – Questionnaire

**Dimension 1 – PARTICIPANT CONTEXT - Sociodemographic profile**

1. Date of Birth: __/__/____

2. Sex:

(a) Female

(b) Male

3. Marital Status:

(a) Single

(b) Married

(c) Divorced

(d) Common-law

(e) Widowed

4. Level of graduate education completed at this institution:

(a) Master of Science (MSc)

(b) Doctorate (PhD)

(c) MSc and PhD

5. Place of Birth (City/State/Country): ____________________.

6. Current Residence (City/State/Country): ____________________.

**Regarding Doctor of Dental Surgery (DDS) degree:**

7. Year of graduation: _____

8. Name of the institution: ____________________.

9. Did you receive a scientific research initiation scholarship during your undergraduate studies in dentistry? (a) Yes (b) No

10. Do you hold any additional undergraduate degrees? (a) Yes (b) No

10.1 If yes, please specify the degree and the year of graduation: ____________________.

11. Are you currently attending or have you completed a Specialization/Residency?

(a) Yes (Specialization)

(b) Yes (Residency)

(c) Yes (Specialization and Residency)

(d) No

11.1 If yes, which area(s)? (Indicate as follows: Specialization or Residency/Area/Year of completion or expected year) ______________________

**Dimension 2 – WORK EXPERIENCE AND PERCEPTIONS REGARDING EDUCATION**

12. BEFORE admission to the graduate program, what was your professional status?

(a) Unemployed

(b) Employed in the public service. What position? ________________

(c) Private service (self-employed)

(d) Private service (employed by others)

(e) Worked as a professor at a Higher Education Institution (HEI)

(f) Other: _______________________

13. DURING your graduate program, you:

(a) Did not work

(b) Employed in the public service.

(c) Private service (self-employed)

(d) Private service (employed by others)

(e) Worked as a professor at a HEI

(f) Started working but stopped before completing the Graduate Program

(g) Started unemployed but finished working

(h) Other: ___________

14. DURING your graduate program, how many hours per week did you dedicate to the program:

(a) Less than 20 hours/week

(b) 20 hours/week

(c) More than 20 and less than 40 hours/week

(d) Full-time (exclusive dedication/40 hours per week)

15. DURING your graduate program, did you participate in research projects with international collaborators linked to the Graduate Program? (a) Yes (b) No

15.1 If yes, which institution and program? ___________

16. DURING your graduate program, did you have interactions with other fields outside Dentistry (*e.g.* research partnerships with other graduate programs)? (a) Yes (b) No
 16.1 If yes, which area(s)? ___________________

17. AFTER completing your graduate program, did you engage in any entrepreneurial activities?

(a) Yes (b) No

17.1 If yes, specify: _____________________________

18. AFTER completing your graduate program, did you pursue a doctoral/post-doctoral program at another institution?

(a) Yes, PhD program only

(b) Yes, post-doctoral program only

(c) Yes, PhD and post-doctoral program

(d) No

18.1 If yes, specify the institution: ____________

19. Regarding your perceptions of the graduate program

For the following questions, please use the scale provided to express your evaluation of the items that describe various aspects of the structure and functioning of the program you completed.

|  | **1** | **2** | **3** | **4** | **5** |
| --- | --- | --- | --- | --- | --- |
| **Conditions and Training Experiences** | Very poor | Poor | Acceptable | Good | Very good |
| Management of the graduate program |  |  |  |  |  |
| Curricular structure |  |  |  |  |  |
| External activities (*e.g.* service) |  |  |  |  |  |
| Opportunities for contact with foreign languages |  |  |  |  |  |
| Infrastructure for teaching and research |  |  |  |  |  |
| Support from the supervisor(s) |  |  |  |  |  |
| Faculty members – profile and experience |  |  |  |  |  |
| Opportunities for exchange nationally |  |  |  |  |  |
| Opportunities for exchange internationally |  |  |  |  |  |

**Dimension 3 – CAREER IMPACT**

20. What is your current employment status?

(a) Student

(b) Unemployed

(c) Employed in public service. What is your position? ________________

(d) Private practice (self-employed)

(e) Private service (employed by others)

(f) Professor at a HEI

(g) Researcher (h) Other:_______________________

21. Have you worked in a field outside of dentistry? (a) Yes (b) No

21.1 If yes, please specify: _________

22. Have you worked as faculty/instructor?

(a) Yes, currently

(b) Yes, in the past

(c) I have never worked as a faculty/instructor

(If not, skip to question 23)

If you have worked as a faculty/instructor:

22.1 As faculty/instructor, what degree(s) did you hold when you started teaching?

(a) MSc

(b) PhD

(c) Post-doctorate

(d) Specialization/Residency

(e) None

22.2 As a faculty/instructor, do/did you teach in the area of your MSc/PhD degree?

(a) Yes

(b) No

(c) Other: ___________

22.3 Do/did you teach at the following levels?

(a) Undergraduate level

(b) Specialization/residency

(c) MSc/PhD

23. Have you held or currently hold any leadership or management roles such as course coordination, committee participation, etc.?

(a) Yes (b) No

23.1 If yes, specify: ____________

24. Impacts of the Graduate Program

For the following questions, please use the 1 to 5 scale below to evaluate each of the following items.

|  | **1** | **2** | **3** | **4** | **5** |
| --- | --- | --- | --- | --- | --- |
| **The impacts of the Program you completed** | Strongly disagree | Disagree | Neutral | Agree | Strongly agree |
| My current employment is related to my graduate degree |  |  |  |  |  |
| The degree was important to improve my wages |  |  |  |  |  |
| The degree allowed me to obtain/change jobs |  |  |  |  |  |
| The program contributed to my professional growth |  |  |  |  |  |
| I feel the results of my work benefited society |  |  |  |  |  |
| The degree allowed me to work with teaching/research |  |  |  |  |  |
| It allowed me to work with education at different levels |  |  |  |  |  |

**Dimension 4 – SCIENTIFIC PRODUCTIVITY**

25. Was your dissertation/thesis published in a scientific journal?

(a) Yes

(b) It is currently under submission/publication process

(c) No

26. CURRENTLY, are you involved a part of a research group?

(a) Yes, as a member of a research group linked to the graduate program I completed

(b) Yes, as a member of a research group at the HEI where I work

(c) Yes, as the leader of a research group at the HEI where I work

(d) Yes, as a member of a research group at a foreign HEI

(e) No, I do not participate in a research group

27. DURING and AFTER your graduate program, what is your research productivity?

(a) Abstract published in national meetings
(b) Abstract published in international meetings
(c) Study published in the proceedings of a national meetings
(d) Study published in the proceedings of an international meetings
(e) Poster presentation
(f) Oral presentation
(g) Participation in symposia
(h) Lecturer role
(i) Article published in national journals
(j) Article published in international journals
(k) Book or book chapter published nationally
(l) Book or book chapter published internationally
(m) Newsletter
(n) Co-publications with industry
(o) Publications cited in patents
(p) Publications with collaborators from other Brazilian institutions
(q) Publications with international collaborators
(r) Technical standards

(s) Patents

(t) Others:____________

**Dimension 5 – FUTURE PERSPECTIVES**

28. Regarding your FUTURE PROJECTS, check the option(s):

( ) Continue my PhD studies in Brazil

( ) Continue my PhD studies abroad

( ) Continue my graduate studies at the post-doctoral level in Brazil

( ) Continue my graduate studies at the post-doctoral level abroad

( ) Apply for a selection process/competition to work as a professor

( ) I intend to continue my teaching activities

( ) I intend to continue my research activities

( ) I do not intend to work as a professor

( ) Other (Specify): ____________________________________

29. Would you recommend the graduate program to a colleague, family member, or acquaintance?

(a) Certainly not

(b) No

(c) Maybe

(d) Yes

(e) Certainly yes

30. Describe how you perceive the impact of the Graduate on your life in:

30.1 personal dimensions

30.2 professional dimension

30.3 academic dimensions
